# Individual differences in selective attention and engagement shape students’ learning from visual cues and instructor presence during online lessons

**DOI:** 10.1038/s41598-023-32069-7

**Published:** 2023-03-28

**Authors:** Jill King, Taylor Marcus, Julie Markant

**Affiliations:** 1 Neuroscience Program, Tulane University, New Orleans, LA 70118 USA; 2 Department of Psychology, Tulane University, 6400 Freret St., 2007 Percival Stern Hall #52, New Orleans, LA 70118 USA; 3 Tulane Brain Institute, Tulane University, New Orleans, LA 70118 USA

**Keywords:** Neuroscience, Cognitive neuroscience, Learning and memory, Psychology, Human behaviour

## Abstract

Although some researchers recommend minimizing extraneous visual information in multimedia lessons, others have demonstrated that features such as visual cues and instructor videos can enhance learning. However, variability in selective attention skills may influence students’ ability to benefit from these additional features. This study investigated links between college students’ selective attention skills and their learning from video lessons that varied in the use of visual cues and the instructor video. Learning outcomes depended on both the visual features available and students’ effort and selective attention skills. Among students who reported increased effort during the lessons, those with more efficient selective attention benefited most when a single additional feature (i.e., either visual cues or the instructor video) was used. All students, regardless of attention skills, benefited when both visual cues and the instructor were combined. These findings suggest that learning during multimedia lessons may depend on the visual features of the lessons and the student’s effort and attention skills.

## Introduction

The dramatic increase in the use of online, multimedia lessons during the COVID-19 pandemic highlighted the importance of research aimed at optimizing these lesson formats. Multimedia lessons typically include static or dynamic pictures (e.g., illustrations, animations, videos) and printed or spoken words (e.g., on screen text, narration^1^). Learning from these lessons can be assessed based on students’ retention, comprehension, or recall of factual knowledge, as well as their ability to match information, solve problems, or transfer information to novel content^2–4^. Although instructors are often advised to minimize the number of extraneous visual features in multimedia lessons to avoid increased cognitive processing demands^5,6^, including some additional visual features in these lessons can facilitate learning (for review see Ref.^7^). For example, the presence of visual cues that direct students’ attention to relevant lesson content reliably benefits learning (e.g., Refs.^8,9^). Several studies have demonstrated that instructor presence during multimedia lessons can similarly facilitate learning^10,11^, but other studies instead found no benefit or a detrimental effect of including the instructor in lessons^12–14^. These mixed results suggest that additional factors shape individual differences in learning during multimedia lessons. In particular, students’ selective attention skills, or their ability to efficiently allocate attention, may impact learning when multiple visual features are included in a lesson. While limited previous research has related individual differences in selective attention to learning outcomes when the instructor was present^15^, it is unknown how selective attention skills affect student learning during lessons that contain multiple additional visual features. To address this question, we assessed college students’ selective attention skills and learning during video lessons that varied based on the presence or absence of visual cues and an instructor video.

Past work aimed at optimizing the design of multimedia lessons has been motivated by the idea that cognitive load, or the amount and nature of information that students process during lessons, can affect learning (for review see Refs.^16,17^). Cognitive load is influenced both by the intrinsic complexity of the learning task and by instructional design factors that shape the extraneous context in which learning occurs (e.g., Refs.^16,18^). For example, college students showed poorer comprehension and transfer of information from lessons that included extraneous text passages and/or images^19^, demonstrating that the presence of irrelevant visual features in multimedia lessons can hinder learning^19–22^. These additional visual features may be detrimental for learning because they capture students’ attention and distract from processing of primary lesson content^23,24^. These findings have led to recommendations that instructors minimize the number of extraneous or irrelevant features in multimedia lessons (e.g., Refs.^5,6^).

**Figure 1:**
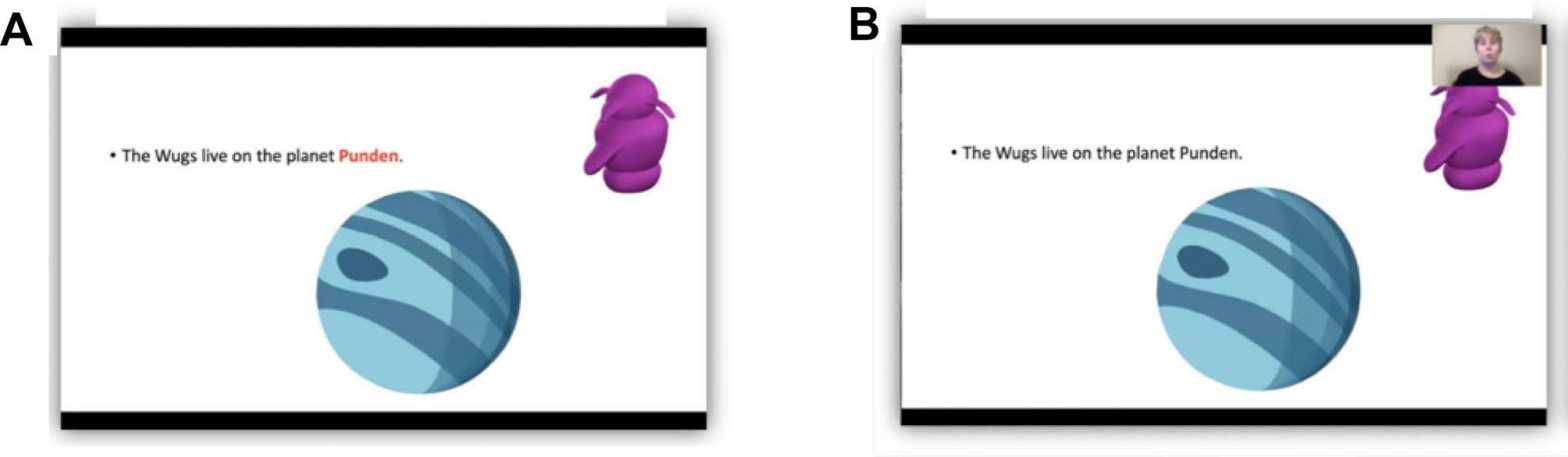
Examples of multimedia lessons with visual cueing and instructor video. Illustration of a multimedia lesson that includes (**A**) visually cued text and (**B**) an instructor video overlay.

However, other studies have found that including additional visual features in multimedia lessons can benefit learning, even though these features may add to students’ cognitive processing load^7,25^. For example, college students retained more information when a lesson narration was accompanied by a subset of the narrated text presented visually^25,26^. Using salient visual cues to direct students’ attention to relevant information (i.e., “signaling”) similarly benefits learning (for review see Refs.^8,9^). Multimedia lessons commonly use arrows, animations, or change the typography or color of the text on the screen to direct students’ attention to relevant content (e.g., see Fig. 1A^27–29^). For example, Ozcelik et al.^29^ cued relevant visual features during a lesson narration by briefly changing the color of labels within corresponding illustrations. These color cues elicited increased looking to the relevant features and enhanced college students’ performance on transfer and matching tests^29^. Although these cues add to the total visual input, they may reduce cognitive load by directing students’ attention to the most relevant information during learning^30,31^.

In addition to visual cues, the presence of the instructor (e.g., via picture-in-picture, lecture capture, or transparent whiteboard presentations; see Fig. 1B) during multimedia lessons can similarly boost college students’ retention and recall of information and their performance on transfer and skill achievement tests^10,11,15,32,33^. This benefit may reflect increased engagement when the instructor is present^34,35^, indicated by students’ effort during learning and their interest in and interaction with the learning material^36,37^. Consistent with this, young adults typically report that they prefer lessons and expect to learn more when the instructor is present^11,38–42^. However, other studies have found that college students showed either no benefit or poorer recall when the instructor was present during multimedia lessons^12,13,39,43^. Several authors have argued that including the instructor only increases extraneous visual features that distract from learning^44,45^. Indeed, unlike visual cues that direct attention to lesson-relevant content, students spend less time looking at primary lesson content when the instructor is present^10,11,13,39,42,46^. Thus, while the presence of the instructor may benefit learning by increasing student engagement, there may also be a cost to learning due to increased processing of extraneous visual features^11,12,33^.

Overall, these findings suggest that including some additional visual features in multimedia lessons can benefit students’ learning. However, while visual cues robustly facilitate learning, the benefits of instructor presence have been less clear. These mixed findings suggest that additional factors may contribute to variability in learning outcomes when multiple features such as visual cues or the instructor video are present. Past research investigating individual differences in learning during multimedia lessons has largely focused on students’ prior knowledge of the lesson topic (see^2,3,16^ for review). For example, visual cues facilitated learning among middle school students who knew less about the lesson topic, but instead interfered with learning for students who had more prior knowledge^47^. Several other factors that may affect these individual differences have been identified (e.g., age, working memory capacity, spatial abilities, motivation/interest in the lesson topic^48–52^), but have not yet been studied in depth^2,3^. As a result, our understanding of the mechanisms that shape individual differences in learning during multimedia lessons remains incomplete.

One possibility is that selective attention skills may influence the extent to which students benefit from visual cues or the instructor video during multimedia lessons. Because cognitive resources are limited, effective learning requires prioritizing processing of information that is the most relevant for ongoing lessons. This attentional prioritization can be mediated by exogenous orienting that drives selection of perceptually salient information (e.g., signaling via a color cue) or by endogenous selective attention mechanisms that allow individuals to focus on task-relevant information while ignoring irrelevant inputs^53,54^. These endogenous selective attention skills are typically assessed via tasks that require individuals to ignore irrelevant stimuli to efficiently focus on a task-relevant target. For example, during the flanker task^55^, participants report the direction of a central arrow while ignoring surrounding arrows that appear in the same direction (congruent condition) or in the opposite direction (incongruent condition). Thus, during the incongruent condition individuals must suppress interference from the surrounding arrows to correctly identify the direction of the target arrow. Individuals who can efficiently engage selective attention to suppress this interference are faster and more accurate to respond during the incongruent condition.

Exogenous orienting skills develop relatively early in childhood^56,57^, whereas endogenous selective attention develops more gradually (e.g., Refs.^58–61^). Although preschool-aged children show expected patterns of responses during the flanker task^62^, performance on this task and related endogenous selective attention tasks continues to improve through childhood and adolescence before peaking in early adulthood (e.g., Refs.^59,61,63,64^). Although both exogenous and endogenous selective attention are mature by early adulthood, individual differences in these attention skills remain throughout adulthood, which may reflect factors such as working memory capacity and task performance strategies^61,65–67^.

These individual differences in selective attention can have important implications for learning. Individuals typically learn less when task-irrelevant (i.e., distracting) information is present in the learning environment^24,68–70^. Individuals with more efficient selective attention can selectively focus on information that is relevant to lessons, whereas those with poorer selective attention can be more susceptible to poor learning outcomes (e.g., Refs.^19,71^). Individuals with more advanced selective attention skills can also more effectively allocate attention to relevant information in the surrounding context (e.g., illustrations) during multimedia lessons^72,73^. These findings suggest that individual differences in selective attention skills may affect how students process additional visual features, such as visual cues and instructor presence, during multimedia lessons. College students showed enhanced retention and transfer of information when visual cues guided their orienting to relevant lesson content more efficiently^29,74^, suggesting that students’ ability to engage selective attention to orient to relevant information may affect the extent to which they benefit from visual cueing. Kokoç et al.^15^ found that college students with more advanced selective attention performed better on a skill achievement test after viewing recorded video lectures that included the instructor, whereas those with poorer attention did not demonstrate this benefit. Overall, these results suggest that students with better selective attention may learn more effectively from multimedia lessons that contain additional visual features, despite the increased cognitive processing load.

In sum, research has shown that extraneous visual inputs in multimedia lessons can impair learning^22,75^, but has also identified visual features that can facilitate learning despite being extraneous to the primary lesson content. Specifically, students show improved learning when visual cues direct their attention to lesson-relevant information^9,29^. The presence of an instructor in multimedia lessons can also improve learning^10,11^, though these effects are less robust^12,14,76^. However, past research has primarily examined effects of visual cues and instructor presence on learning independently, even though multimedia lessons commonly incorporate multiple visual features concurrently. As a result, the extent to which these features interact to influence learning is less clear. Johnson et al.^77^ found that middle-school students performed better on problem-solving tests following lessons that included two forms of visual cues (i.e., arrows and an animated pedagogical agent) that directed students’ attention to relevant information. These results suggest that combining visual cues and instructor presence may provide additional benefits for learning. Alternatively, past research indicating that the instructor can capture attention and distract from other lesson elements^11,13,39^ suggests that students may have more difficulty efficiently allocating attention when both visual cues and the instructor are present. Finally, while limited research suggests that students’ selective attention skills may influence the extent to which they benefit from visual cues or instructor presence^15^, it is unknown how selective attention affects learning during more complex multimedia lessons that combine these visual features.

**Figure 2:**
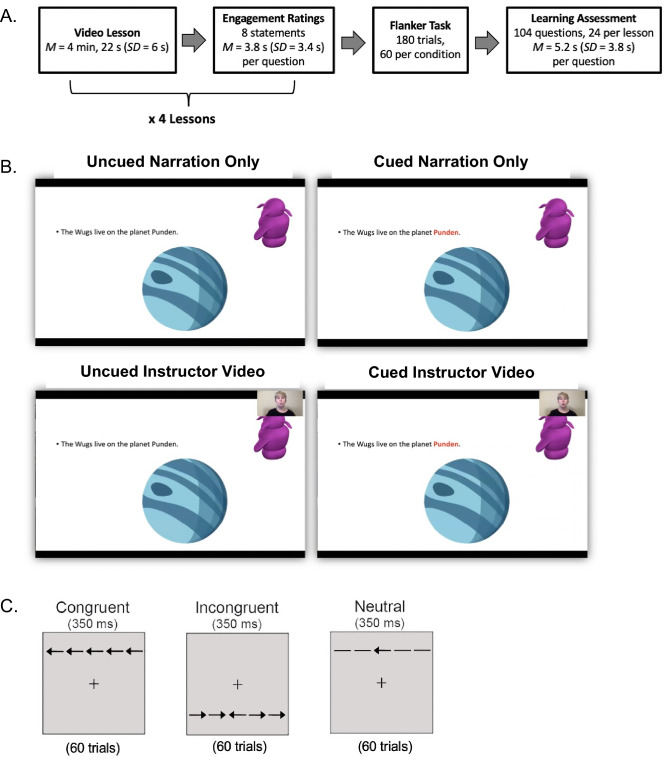
Schematic illustration of the learning and selective attention tasks. (**A**) Timeline of the learning and selective attention tasks; (**B**) illustration of the four lesson conditions presented during the learning task; and (**C**) illustration of the three trial types presented during the flanker selective attention task.

To address these questions, we examined college students’ learning from video lessons that varied in their use of visual cues and an instructor video. Figure 2 illustrates the study design. Students viewed pre-recorded video lessons that (1) included only the instructor’s narration and the slide presentation (Uncued Narration condition), (2) additionally included visual cues in which the most relevant text appeared in bold, red font (Cued Narration condition), (3) included the instructor video but no visual cues (Uncued Instructor Video condition), and (4) included both the visual cues and instructor video (Cued Instructor Video condition). Students additionally rated their effort and engagement during the video lessons and completed a flanker task as an independent measure of selective attention. We assessed the relation between students’ reported effort during the lessons, individual differences in selective attention performance, and the extent to which students benefited from the additional visual features during the video lessons. We expected that the visual cues and instructor video would facilitate learning, consistent with prior research^9–11,29^. However, we also expected that these effects would depend on students’ selective attention skills. Specifically, we predicted that individuals with poorer selective attention skills may have more difficulty effectively allocating attention when multiple additional visual features were included in a single lesson. In contrast, we expected that students with better selective attention skills may distribute their attention more effectively across the primary lesson content and multiple visual features and therefore benefit from the presence of these additional features to a greater extent.

## Results

### Preliminary analyses

Preliminary bivariate correlations are reported in Supplementary Table 1. Satisfaction, efficacy, engagement, and effort ratings were highly correlated (r’s > 0.41, p’s < 0.001). Participants who indicated that the lessons were more difficult reported increased engagement (r = 0.20, p = 0.044) and reduced efficacy (r =  − 0.22, p = 0.024). Difficulty ratings were unrelated to satisfaction or effort (p’s > 0.3). We therefore focused only on effort and difficulty ratings for ease of interpretation. Effort ratings were related to overall learning scores (r = 0.31, p = 0.001), but difficulty ratings were not (p = 0.194). There was also a trend-level correlation between RT cost scores and overall learning scores (r =  − 0.17, p = 0.09), indicating that participants with poorer selective attention skills tended to learn less across all lesson conditions. Finally, participants’ effort and difficulty ratings did not vary across the lesson conditions (p’s > 0.2). See Supplementary Results for details.

### Learning outcomes

Participants showed overall moderate accuracy during the post-lesson assessments (M = 0.65, SD = 0.19). Performance on the learning assessment was reliably above chance (i.e., 0.25) for all lesson topics (p’s < 0.001), indicating that participants successfully learned the material presented in all video lessons.

We next examined the extent to which learning from the video lessons was influenced by visual features of the learning task (i.e., visual cues, instructor video), selective attention skills, and perceived effort during the lessons. We examined accuracy on the learning assessments using a repeated measures ANCOVA with instructor presence (narration only, instructor video) and cueing (uncued, cued) as within-subjects factors. We included centered RT cost scores, centered effort ratings, and the RT cost x effort interaction term as covariates.

**Figure 3:**
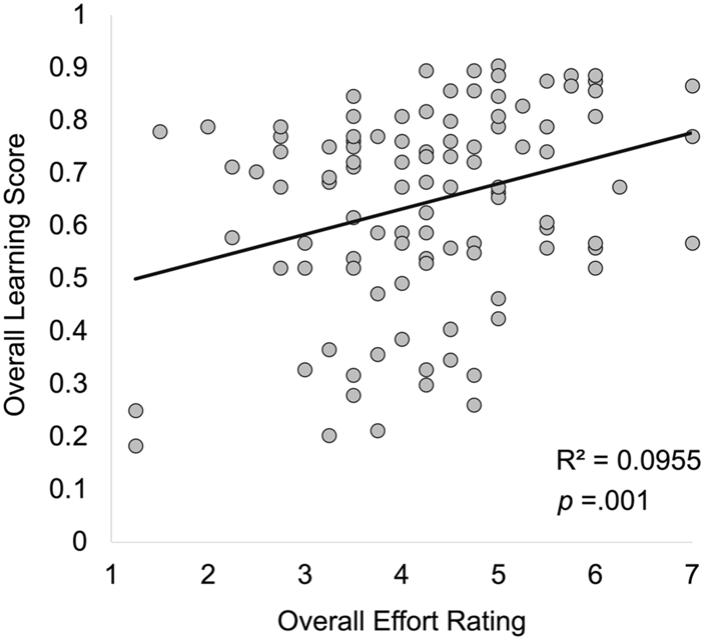
Relation between perceived effort and overall learning outcomes.

ANCOVA results indicated a main effect of perceived effort, F(1, 103) = 9.11, p = 0.003, η_p_^2^ = 0.08; Fig. 3. Consistent with the preliminary results above, participants who reported increased effort during the lessons performed better on all learning assessments (r = 0.29, p = 0.002). The main effect of RT cost scores was not significant (p = 0.19).

**Figure 4:**
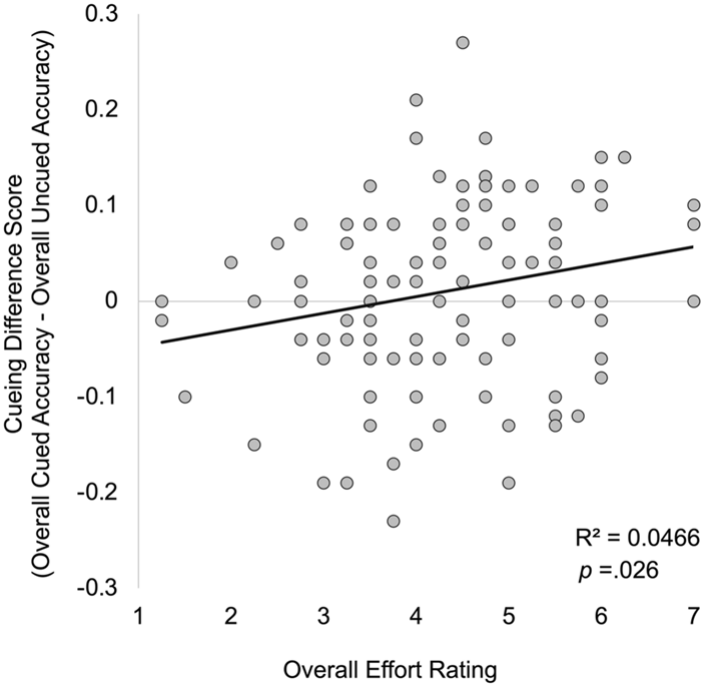
Relation between perceived effort and learning during cued vs. uncued lessons. Participants who reported higher effort learned more from lessons that included visual cues compared to those that did not.

Results also indicated a cueing x perceived effort interaction, F(1, 103) = 4.01, p = 0.048, η_p_^2^ = 0.04. Participants who reported higher effort showed overall higher learning scores for both the uncued (r = 0.25, p = 0.01) and the cued lessons (r = 0.35, p < 0.001). To further examine this interaction we computed a difference score by subtracting each participant’s overall accuracy during uncued lessons from their overall accuracy during cued lessons. More positive difference scores indicate improved learning during cued lessons compared to uncued lessons. Participants who reported overall higher effort learned more during cued lessons compared to uncued lessons (r = 0.22, p = 0.026; Fig. 4).

Finally, results indicated a cueing × instructor presence × perceived effort interaction, F(1, 103) = 4.54, p = 0.035, η_p_^2^ = 0.04, which was further moderated by a significant four-way cueing x instructor presence × RT cost × perceived effort interaction, F(1, 103) = 5.04, p = 0.027, η_p_^2^ = 0.05. To further explore this four-way interaction, we computed difference scores to determine the extent to which each participant’s learning changed as a function of the additional visual features. We computed a Cueing Benefit score based on the difference in performance during the Cued Narration vs. the Uncued Narration conditions, an Instructor Benefit score based on the difference in performance during the Uncued Instructor Video vs. Uncued Narration conditions, and a Full Features Benefit score based on the difference in performance during the Cued Instructor Video vs. Uncued Narration conditions. Higher scores indicated an increased learning benefit when the additional visual features were added.

**Figure 5:**
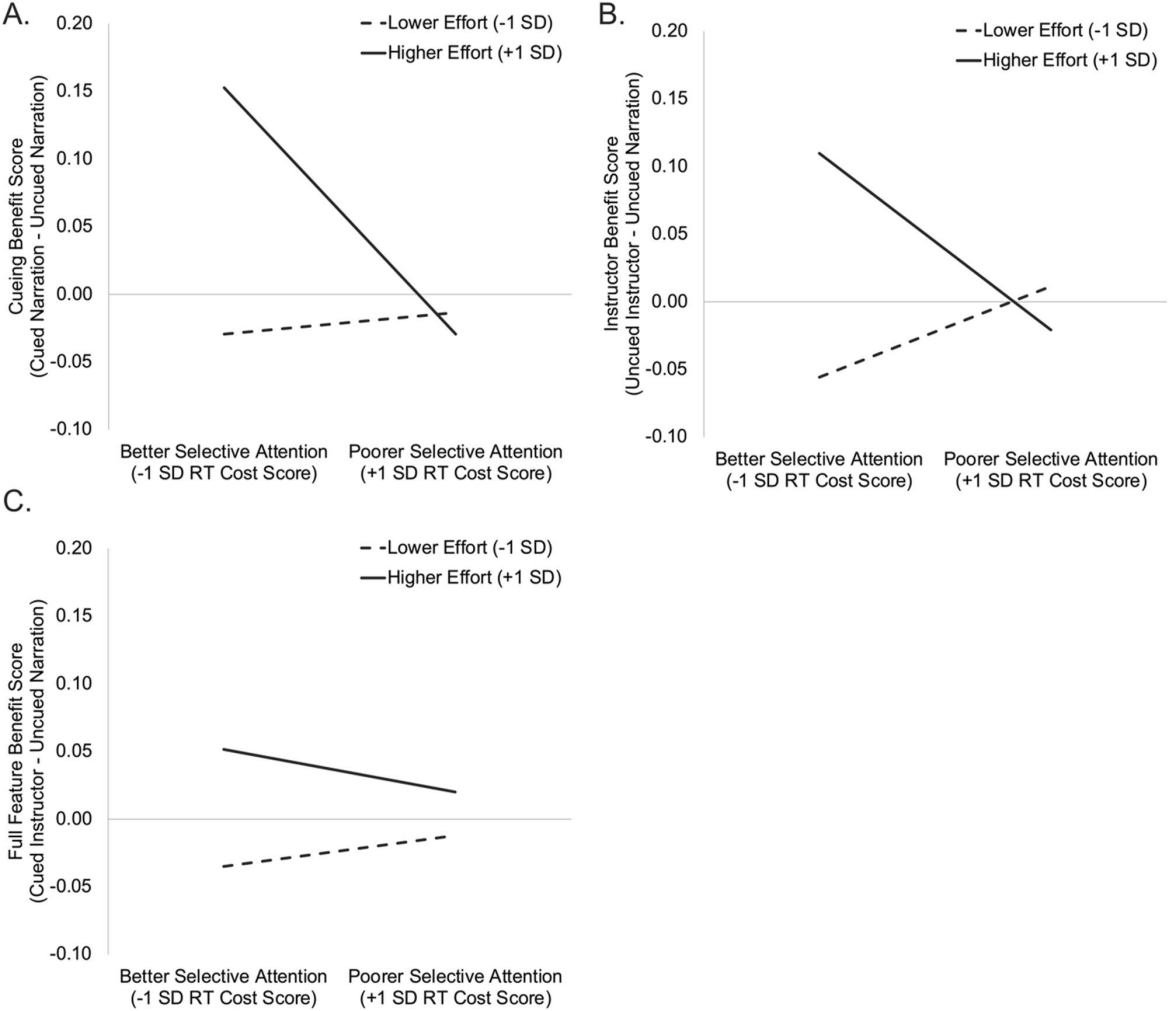
Simple slopes illustrating the interaction between selective attention and perceived effort on learning improvements in the presence of visual cues and/or the instructor video. Among participants who reported higher effort, those with better selective attention (i.e., lower RT cost scores) benefited more from the addition of only the (**A**) visual cues or (**B**) the instructor video. However (**C**) selective attention skills did not moderate learning improvements when both visual cues and the instructor video were included in the lessons.

Follow-up analyses indicated a significant RT Cost x perceived effort interaction for the Cueing Benefit score, b =  − 0.001, t(103) =  − 2.29, p = 0.024, and a trend-level RT Cost × perceived effort interaction for the Instructor Benefit score, b =  − 0.001, t(103) =  − 1.72, p = 0.088. We used simple slopes analyses to examine the moderating effects of perceived effort on the relation between selective attention skills (i.e. RT cost scores) and Cueing Benefit scores. Among participants who reported higher effort during the lessons (i.e., effort ratings 1 SD above the group mean), there was a significant negative relationship between RT cost scores and Cueing Benefit scores, b =  − 0.002, t(103) =  − 3.70, p < 0.001; Fig. 5A. Results similarly showed a significant negative relationship between RT cost scores and the Instructor Benefit score among participants who reported higher effort, b =  − 0.002, t(103) =  − 2.52, p = 0.013; Fig. 5B. For these higher-effort participants, better selective attention skills (i.e., lower RT cost scores) were associated with an increased benefit from the addition of either the visual cues or the instructor video. In contrast, for participants who reported lower effort (i.e., effort ratings 1 SD below the group mean), selective attention skills were unrelated to learning benefits from either the visual cues or the instructor video (p’s > 0.11). Finally, the RT Cost × perceived effort interaction was unrelated to the Full Features Benefit score (p = 0.389; Fig. 5C), indicating that selective attention skills did not influence learning improvements when both visual cues and the instructor video were included in the lesson.

## Discussion

We investigated the extent to which individual differences in selective attention skills influenced college students’ learning when additional visual features (i.e., visual cues, instructor video) were present during online video lessons. Students viewed pre-recorded video lessons that varied based on the presence of (1) visual cues highlighting relevant lesson content and (2) a video overlay of the instructor. Participants also completed a flanker task as an independent measure of selective attention skills and reported their perceived effort during the video lessons. Learning outcomes were influenced by the visual features within the video lessons as well as individual differences in students’ reported effort during the lessons and their selective attention skills. Students who reported increased effort learned more overall and specifically learned more from lessons with visual cues. Furthermore, among these students who reported higher effort, those who demonstrated more efficient selective attention benefited most from either visual cues or the instructor video. Because the observed effect sizes are moderate, it will be important for future research to both replicate and extend these findings. Nonetheless, the current results suggest that learning during multimedia lessons may depend on the visual features within the lesson and individual differences in students’ effort and attention skills.

The current study demonstrated a link between students’ perceived effort during the video lessons and enhanced learning, which is consistent with past research indicating that student engagement in the classroom relates to both motivation^78,79^ and learning outcomes^80–82^. Student engagement can be divided into cognitive, emotional, behavioral, and agentic components^36,83,84^. The measure of perceived effort in the current study overlaps most closely with behavioral and cognitive dimensions of engagement, which are typically assessed based on attentiveness, persistence, degree of effort, and psychological investment^85,86^. Both behavioral and cognitive engagement have been linked to enhanced learning^87,88^, but emotional and agentic dimensions of engagement also facilitate learning^84,89^. Although we focused on effort ratings in our analyses, students’ reported effort was also correlated with ratings of learning efficacy, satisfaction, and engagement with the lessons. Thus, it is possible that there is a more global impact of engagement on student learning outcomes that is not specific to a single dimension.

Among students who reported higher effort, the presence of visual cues facilitated learning, consistent with past studies demonstrating that visual cues both increase attention to lesson content and improve learning^8,9,29,74,90^. Consistent with this, students in the current study learned more when visual cues were added to the lesson, even when the instructor video was not included. In the current study we used typography and color cues (i.e., bold, red font), which we selected based on past studies (e.g., Refs.^29,74,91^). However, multimedia lessons can include a range of other visual cues (e.g., animations, arrows^9^) and additional work is needed to determine whether the observed links between student effort, selective attention skills, and learning benefits from visual cues generalize across this broader array of cue types. Nonetheless, we observed an effect of cueing despite other design differences across the current and previous studies (e.g., lesson duration, novel content), which provides further evidence that the presence of visual cues reliably facilitates learning during multimedia lessons.

Previous work examining effects of instructor presence during multimedia lessons has yielded more mixed results. Several studies have found that the presence of the instructor facilitates students’ learning^10,11,15,32,33^, while others suggest that including the instructor has no benefit or may be detrimental for learning^12–14,39,43^. In the current study we did not find an overall effect of instructor presence on students’ learning outcomes. Although these results do not align with past work indicating that the instructor video boosts learning, they converge with a recent meta-analysis that found no overall benefit of instructor presence on learning^76^. Researchers have questioned whether the instructor video is an extraneous visual feature that may hinder learning by drawing attention away from primary lesson content^6,19,45^ or if the presence of the instructor instead provides salient social cues that can increase student engagement during learning^10,76,92^. We found that perceived effort and difficulty ratings did not vary across lessons, suggesting that including the instructor video did not substantially impact engagement. However, in the current study the instructor used a neutral tone, maintained a neutral gaze, and avoided using gestures throughout the lessons. Although these neutral features were intended to maintain consistency across lessons, they may have dampened participants’ engagement. It remains possible that the instructor video may have had a stronger overall learning benefit if it included features that were more socially salient (e.g., verbal cues, eye gaze, facial expressions, gestures^93–95^). Future research will be needed to determine how varying these features within the instructor video affects students’ attention, motivation, and learning at the both the group and individual level.

Although there was no effect of instructor presence at the group level, the extent to which students benefited from the visual cues or the instructor video depended on individual differences in selective attention skills and effort during the lessons. Among participants who reported higher effort during the lessons, those with more advanced selective attention skills benefited more from either visual cues or the instructor video, relative to their performance during the narration only condition in which these features were absent. This result is consistent with evidence that more advanced attention skills facilitated learning from lessons that required integrating information from multiple sources (i.e., text and corresponding illustrations^72^). King and Markant^73^ similarly found that children with more advanced selective attention looked longer at illustrations that were relevant to ongoing lessons, which in turn facilitated learning. Conversely, individuals with poorer selective attention learned less from video lessons that included the instructor video and looked longer at the instructor during these lessons^15^, suggesting that students with inefficient selective attention may have more difficulty attending to primary lesson content when the instructor is present. Although attention control is most often defined as the ability to focus on a target while ignoring irrelevant information (e.g., Ref.^96^), efficient attention control also involves flexibly shifting attention across task-relevant features or inputs^97,98^. The current findings suggest that more advanced selective attention skills may facilitate learning by allowing individuals to efficiently allocate attention across multiple relevant features that are available during multimedia lessons.

To our knowledge, this is the first study to examine both visual cues and the instructor video during the same lessons. At the group level there were no differences in learning across lesson conditions, suggesting that combining the visual cues and the instructor video did not facilitate learning beyond presenting either of these features alone. We also found that students’ selective attention skills did not relate to learning during lessons that contained both visual cues and the instructor video. Instead, the combined presence of these visual features benefited learning to a similar extent for all participants who reported higher effort during the lessons, regardless of their selective attention skills. This contrasts with the lessons that included only one additional visual feature (i.e., visual cues or the instructor video), in which selective attention skills had a stronger effect on learning outcomes. These results may suggest that the combination of visual cues and the instructor video benefits learning more generally after accounting for individual differences in selective attention skills.

The current study extends previous work identifying additional visual features that benefit learning despite increased cognitive processing demands^7^. Like this previous research, we found that students learned more when additional features were available. However, the extent of this benefit depended on individual differences in students’ selective attention skills and effort during learning. These results suggest that the same lesson design may not be equally beneficial for all students. Future research will be needed to determine how to optimize the design of multimedia lessons while accounting for these individual differences in attention and engagement. Our results suggest that students with more advanced selective attention who are engaged in ongoing lessons may benefit most from additional visual features, as they can efficiently allocate attention across these features and primary lesson content. However, the current results also suggest that combining visual cues and the instructor video may be optimal when individualized designs are not possible, since all students benefited from this approach when they were engaged with the lesson. It is also important to note that the current study only used relatively short, pre-recorded video lessons. Although this design aligns with the recommended duration for online instructional videos^99^, it is possible that selective attention skills may differentially impact learning during shorter or longer lessons. Furthermore, it is unclear whether the current results will generalize to other learning contexts, such as synchronous online or traditional classroom settings, that typically afford more opportunity for contingent interaction with the instructor and classmates and a wider range of contextual information (e.g., classroom decorations). Future research can further investigate how individual differences in selective attention and student engagement affect learning in these dynamic contexts. Finally, while this study focused on visual features, multimedia lessons require students to integrate both auditory and visual inputs. Additional research is needed to relate individual differences in selective attention to this multimodal processing, which will be especially important for understanding how learning from multimedia lessons may vary for different populations of students. For example, while participants in the current study had normal hearing and vision, other students may have sensory processing limitations that affect the extent to which they rely on vision or hearing during multimedia lessons. Future research can investigate how selective attention skills affect these students’ processing of complex multimedia lessons.

## Conclusions

The current study demonstrated that students who reported increased effort learned more overall and especially benefited from video lessons that contained perceptually salient visual cues. However, students’ learning was also shaped by individual differences in their selective attention skills. Among those who reported higher effort, students who also demonstrated more advanced selective attention benefited more from the presence of either visual cues or the instructor video. Combining these visual features benefited learning to a similar extent for all students who were engaged in the lesson, regardless of their attention skills. Individual learning outcomes during multimedia lessons may therefore depend on interactions between both individual factors (e.g., engagement, selective attention skills) and extrinsic factors (e.g., the extent to which relevant visual features are available in the learning environment).

## Methods

### Participants

One-hundred and seven adults (81 F; M_Age_ = 21.5 years, SD = 3.0 years) completed the study. We determined sample size based on a power analysis (power = 0.9, alpha = 0.05) which indicated that a minimum of N = 75 would be needed to detect effects similar to those observed previously^15,29,42^. Based on self-report, 66.4% of participants were White/Caucasian, 9.3% were Multiracial, 9.3% were Asian, 6.5% were Hispanic, 4.7% were Black, and 3.7% identified as Other. All participants had normal or corrected-to-normal hearing and vision, according to self-report. Nine additional participants were excluded from analyses because they did not complete both the learning and selective attention tasks (N = 6), their selective attention scores were > 2 SD above or below the group mean (N = 2), or due to technical error (N = 1). Because the test session was fully remote, all participants provided informed eConsent via REDCap^100^. Participants received a $15 gift card or academic credit after completing the study. All study protocols were approved by the Tulane University Institutional Review Board. All study methods were carried out in accordance with relevant guidelines and regulations.

### Materials and procedure

Participants completed a fully remote test session via video conference between December 2020 and March 2021, during which they completed online learning and selective attention tasks hosted via Pavlovia.org (see Fig. 2A for the order of task completion). We used PsychoPy3^101^ to present the tasks in a full-screen web browser. The tasks required approximately 40 min to complete. We report all stimuli dimensions in percentage of the screen since we did not have detailed information about participants’ computer displays. Participants did not see the experimenter during the task but could communicate with them verbally. We confirmed the validity of these online methods by conducting an initial pilot study (N = 25, M_Age_ = 20 years, SD = 1.5 years), which replicated effects typically observed with flanker tasks in laboratory settings^55,63^ (see Supplementary Materials).

#### Learning task

Participants first completed a learning task in which they viewed four pre-recorded video lessons that conveyed novel content about the lives of fictional alien families (see Supplementary Materials). We developed novel lesson content to control for baseline subject knowledge^73,77,91^. Participants in the pilot study (N = 25) showed successful learning but below ceiling performance (M_Accuracy_ = 0.75, SD = 0.11; see Supplementary Materials) confirming that the lesson content was appropriately difficult.

Each lesson consisted of a pre-recorded slide presentation that included text, images, and narration by an instructor. Across lessons we manipulated the presence vs. absence of visual cues highlighting lesson-relevant text and the instructor video. For lessons with visual cues, the most relevant lesson text appeared in bold, red text as the instructor discussed it. We selected these typography and color cues based on previous research that identified benefits of visual cueing during multimedia lessons (e.g., Ref.^90^). We determined text relevance based on how closely it matched the correct answer for the corresponding assessment question (see Supplementary Table 2). For lessons that included the instructor video, we recorded the slide presentations with a picture-in-picture video overlay of the instructor in the top right corner of the display. The instructor video overlay constituted 2.6% of the display (11.8 cm × 6.6 cm on a 13-in. laptop). The same female instructor used a neutral tone to deliver all lessons. In all video recordings, the instructor appeared against a neutral background with their face and shoulders visible. The instructor’s clothing and accessories were identical across lessons and the instructor maintained a neutral facial expression and looked directly at the camera throughout each lesson.

Participants viewed the video lessons across four conditions (Fig. 2B). During the Uncued—Narration condition, participants saw the slides and heard the instructor’s narration. The Cued—Narration condition was similar but also included the visual cues to direct attention to relevant text. During the Uncued—Instructor Video condition participants saw the slides and the picture-in-picture overlay of the instructor video. Finally, the Cued—Instructor Video condition included both the visual cues and the instructor video. We developed 16 pre-recorded video lessons, with one video for each of the four lesson topics across each of the four lesson conditions. Because the videos were dynamic, lesson duration varied slightly across conditions (M = 4 min, 22 s; SD = 6.0 s; see Supplementary Table 3). The lesson phase comprising the four lesson conditions lasted 18 min.

At the beginning of the learning task participants were told that they would view four video lessons about the lives of alien families and answer questions about each video. They were not told when they would be tested. Initial analyses indicated that participants learned more from the first lesson compared to all others (p’s < 0.001, d’s > 0.4) but there were no differences across the remaining lessons (p’s > 0.08). We counterbalanced the order of lesson conditions and the pairing of lesson topic and condition across participants to ensure that observed effects of lesson condition could not be attributed to this primacy effect. We also pseudorandomized the order of lesson topics to ensure that all possible stimuli combinations were evenly distributed across participants.

#### Engagement ratings

After each video, participants rated their perceived difficulty of the lesson, their level of engagement and effort invested in learning the content, their satisfaction with the lesson, and their perceived learning efficacy. We developed the rating scales based on research that used similar measures to evaluate students’ satisfaction, motivation, and engagement during video lessons^38,46^. Participants used a 1 to 7 Likert scale to respond to each statement (see Supplementary Table 4). Each statement appeared in black text on a gray background with an illustration of the 1–7 scale included to anchor the ratings. Participants required 2 min to complete the engagement ratings for all lessons.

#### Selective attention task

After viewing all four video lessons participants next completed the flanker selective attention task. Stimuli included a central target arrow and two distractor arrows on the left and right sides of the target. The distractor arrows surrounding the central target appeared either in the same direction as the central arrow (congruent condition), in the opposite direction (incongruent condition), or as horizontal lines without directionality (neutral condition; Fig. 2C). This resulted in a single row of five arrows that constituted 0.05% of the display, (i.e., 3 cm on a 13-inch laptop). The target and distractor arrows appeared 1 cm either above or below a central fixation cross. All stimuli were black images on a gray background.

Each trial began with presentation of the central fixation for a variable duration (1000, 1500, 2000, or 2500 ms) before the arrows appeared above or below the fixation for 350 ms. Participants could respond within 1700 ms after stimulus onset. Participants were instructed to focus on the central fixation and use key presses to indicate the direction of the central arrow (“J” = left; “K” = right). Participants completed 12 practice trials with audiovisual feedback and then completed 180 trials (60 per trial type) presented in random order, which required approximately 10 min to complete. Each trial type had an equal number of trials with arrows above and below the central fixation and with the central arrow pointing to the left and right.

#### Learning assessment

Finally, participants completed the learning assessment for all four video lessons after the flanker task. Participants answered 104 multiple-choice questions (26 for each lesson topic), which required approximately 10 min to complete. Each question mapped onto a single fact presented during the video lessons (see Supplementary Table 2). Each question and four answer choices appeared as black text against a gray background and remained visible up to 47 s. Participants used their keyboard to respond (M = 5.20 s, SD = 3.82 s), after which the trial advanced. Participants completed all questions in a single block in random order. Answer choices were pseudorandomized such that the correct answer (1, 2, 3, or 4) occurred the same number of times across all questions and for each lesson topic.

### Data processing

#### Learning and engagement outcomes

For each lesson condition we computed an overall engagement score by averaging the ratings for the three engagement statements^38^. We measured learning outcomes based on accuracy during the post-lesson assessment.

#### Individual differences in selective attention

We examined performance on the flanker task as a measure of individual differences in selective attention. We computed accuracy based on the proportion of trials in which the participant correctly identified the direction of the central arrow. We also computed average response times (RT), excluding trials with inaccurate responses or those in which the response time was > 2 SD above the individual mean.

Initial analyses indicated that participants were slower and less accurate to indicate the direction of the central arrow during the incongruent condition compared to both the congruent and neutral conditions (p’s < 0.001, d > 0.7; see Supplementary Materials). To assess individual differences in selective attention, we computed RT cost scores for each participant (i.e., incongruent RT–congruent RT). Reduced RT costs during the incongruent condition indicate more efficient selective attention, as individuals can more quickly suppress the surrounding distractors to respond to the central target arrow. Conversely, higher RT cost scores indicate increased difficulty filtering conflicting information.

## Supplementary Information

Supplementary Information.

## Data Availability

The datasets generated during the current study are available from the corresponding author on reasonable request.
