# Supplementary material for: Individual differences in selective attention and engagement shape students’ learning from visual cues and instructor presence during online lessons
Source: Sci Rep. 2023 Mar 28;13:5075. doi: 10.1038/s41598-023-32069-7 (PMC10047463; doi:10.1038/s41598-023-32069-7)

**Supplementary Results: Primary Study**

**Selective Attention Task**

Initial analyses indicated that participants showed the expected selective attention responses at the group level. Participants showed overall high accuracy in determining the directionality of the target arrow (M = .95, SD = .06). We examined participants’ accuracy and RT during the flanker task using repeated-measures ANOVA with trial type (congruent, incongruent, neutral) as the within-subject factor. We used Greenhouse-Geisser corrections to account for violations of sphericity. Results indicated a main effect of trial type on accuracy, F(1, 108.2) = 45.52, p < .001, η_p_^2^ = .30. Participants were significantly less accurate during incongruent trials (M = .86, SD = .19) compared to both congruent and neutral trials (M_Congruent_ = .98, SD = .03; t(106) = 6.81, p < .001, d = .95; M_Neutral_ = .98, SD = .04; t(106) = 6.73, p < .001, d = .95). Participants were also marginally more accurate during congruent trials compared to neutral trials (t(106) = 1.99, p = .049, d = .20).

A second ANOVA similarly revealed a main effect of trial type on RT, F(1.3, 137.2) = 630.88, p < .001, η_p_^2^ = .86; Supplementary Figure 1. Participants were significantly slower to respond to the central target during incongruent trials (M = 691.32 ms, SD = 85.63 ms) compared to both congruent and neutral trials (M_Congruent_ = 587.85 ms, SD = 74.19 ms; t(106) = 25.75, p < .001, d = 2.58; M_Neutral_ = 577.23 ms, SD = 74.67 ms; t(106) = 26.70, p < .001, d = 2.66). Participants were also slower to respond during congruent trials compared to neutral trials (t(106) = 5.79, p < .001, d = .56). Overall, these results replicate previously established findings demonstrating performance costs when participants are required to suppress competing spatial information during incongruent trials^46,76^.

**Preliminary Analyses**

Preliminary bivariate correlations are reported in Supplementary Table 1. Results indicated positive correlations across participants’ satisfaction, efficacy, engagement, and effort ratings (r’s > .41, p’s < .001). There was a significant positive relation between overall effort ratings and overall learning scores (r = .31, p = .001), as well as a trend-level significant negative correlation between RT cost scores and overall learning scores (r = -.17, p = .09), indicating that participants with higher RT cost scores (i.e., poorer selective attention skills) tended to learn less across all four lesson conditions. Difficulty ratings related to efficacy and engagement (r_Efficacy_ = -.22, r_Engage_ = .20, p’s < .05) but were unrelated to satisfaction or effort (p’s > .3). Difficulty ratings were also unrelated to RT cost scores or overall learning scores (p‘s > .19). Participant age was marginally related to overall learning scores (r = .19, p = .051) but was unrelated to RT cost scores or overall effort ratings (p’s > .12). Finally, independent samples t-tests indicated that participant gender was unrelated to overall learning scores, RT cost scores, or overall effort ratings (p’s > .2).

We also conducted initial analyses to determine whether participants’ perceived effort or difficulty ratings varied across lesson conditions. We examined these ratings using repeated measures ANOVAs with instructor presence (narration only, instructor video) and cueing (uncued, cued) as within-subjects factors. Results indicated that there were no significant effects of instructor presence, cueing, or instructor presence x cueing interaction for either the perceived effort or difficulty ratings (p’s > .20).

**Supplementary Methods: Primary Study**

**Learning task.** Participants first completed a learning task in which they viewed four pre-recorded video lessons that conveyed novel content about the lives of fictional alien families. Supplementary Table 2 describes the content for each lesson topic. (see Supplementary Materials for details). See https://osf.io/4fr6s/?view_only=1e272c280261410fbbdbe5fb6dad5a12 to view the lesson videos in full.

**Supplementary Results: Pilot Study**

**Learning task**

Participants showed overall moderate accuracy in responding to the post-lesson assessment questions (M = .75, SD = .11), which was unrelated to gender (p = .373) or age (p = .411). One-sample t-tests indicated that performance on the learning assessment was reliably above chance (i.e., .25) for all lessons (M_Wugs_ = .77, SD = .13; M_Bliffs_ = .77, SD = .13; M_Chuttles_ = .72, SD = .14; M_Merbas_ = .75, SD = .15; p’s < .001). Paired t-tests indicated that participants learned less from the Merbas video lesson compared to the Wugs video lesson (t(24) = 2.14, p = .043), but there were no other differences in performance across video lessons. This difference was unlikely to affect learning across lesson conditions in the primary task described in the main manuscript because lesson content and order were counterbalanced.

**Selective Attention Task**

Participants showed overall high accuracy in determining the directionality of the target arrow (M = .97, SD = .04). Participant gender and age were unrelated to accuracy and response times during the flanker task (p’s > .13). Repeated measures ANOVA with Greenhouse-Geisser corrections indicated a main effect of trial type on accuracy, F(1.1, 26.8) = 15.73, p < .001, η_p_^2^ = .40. Participants were significantly less accurate during incongruent trials (M = .91, SD = .12) compared to both congruent and neutral trials (M_Congruent_ = .98, SD = .05; t(24) = 4.20, p < .001, d = .82; M_Neutral_ = .98, SD = .04; t(24) = 4.20, p = .001, d = .78). There was no difference in accuracy across congruent and neutral trials (p = .709).

A repeated measures ANOVA similarly revealed a main effect of trial type on RT, F(1.1, 29.6) = 123.9, p < .001, η_p_^2^ = .84. Participants responded more slowly to the central target during incongruent trials (M = 687.98 ms, SD = 104.46 ms) compared to both congruent and neutral trials (M_Congruent_ = 577.68 ms, SD = 61.94 ms; t(24) = 10.63, p < .001, d = 1.33; M_Neutral_ = 565.79 ms, SD = 67.78 ms; t(24) = 12.46, p < .001, d = 1.42). Participants also responded more slowly during congruent trials compared to neutral trials (t(24) = 2.98, p = .007, d = 0.19). Overall, these results replicate previously established findings demonstrating performance costs when participants are required to suppress competing spatial information during incongruent trials^46,76^. This successful replication confirms the validity of the remote data collection methods used in the current study.

**Supplementary Methods: Pilot Study**

We conducted an initial pilot study to confirm the validity of remote data collection methods and ensure that lesson content was appropriately difficult for our participant group.

**Participants**

Twenty-five adults (18 F; M_Age_ = 21.4 years, SD = 1.6 years) completed the pilot study. Sixty percent of participants reported that they were White/Caucasian, 20% were Asian, 12% were Multiracial, and 8% were Hispanic. All participants provided informed consent and received a $15 gift card or academic credit for their participation.

**Materials and Procedure**

Participants completed a fully remote test session, as described in the main manuscript, comprised of a learning task followed by the flanker selective attention task.

**Learning task.** During the learning task participants viewed four pre-recorded video lessons that conveyed novel content about the lives of fictional alien families (see Supplementary Table 2). At the beginning of the learning task, participants were told that they would view four video lessons about the lives of alien families and answer questions about each video. During the pilot study every lesson consisted of a pre-recorded slide presentation that included text, images, and narration by an instructor. None of the lessons included visual cues or the instructor video. Because the video lessons were dynamic, the duration of the videos varied slightly across lesson topics (e.g., due to small differences in the number of spoken words per lesson; see Supplementary Table 3).

Participants completed the learning assessment for all four video lessons after viewing the final lesson (N = 19) or after finishing the flanker task (N = 6). Participants completed 104 multiple-choice assessment questions (26 for each lesson topic), each of which mapped onto a single fact presented during the lessons (see Supplementary Table 2). Each question and four answer choices appeared as black text against a gray background and remained visible up to 47 s, including 2 s to initially read the question and answers followed by a 45 s response period. Participants used their keyboard to make a response (1, 2, 3, or 4) and trials advanced when the participant responded. Participants completed all questions in a single block in random order. Answer choices were pseudorandomized such that the correct answer occurred the same number of times across all questions and for each lesson topic.

**Selective attention task.** Pilot study participants also completed a flanker selective attention task that was identical to the task described in the main manuscript. Stimuli included a central target arrow and two distractor arrows to the left of the target and two distractor arrows to the right of the target. The distractors flanking the central target appeared in the same direction as the central arrow (congruent condition), in the opposite direction (incongruent condition), or as horizontal lines without any directionality (neutral condition). Each trial began with presentation of the central fixation for a variable duration (1000, 1500, 2000, or 2500 ms) before the arrow stimuli appeared above or below the fixation point for 350 ms. Participants used key presses to indicate the direction of the central arrow (“J” = left-facing arrow; “K” = right-facing arrow) and had 1700 ms after stimulus onset to make a response. Participants completed 12 practice trials with audiovisual feedback, and then completed 180 trials (60 per trial type) presented in random order. Each trial type had an equal number of trials with arrows above and below the central fixation point and with the central arrow pointing to the left and right.

**Data Processing**

We measured learning outcomes based on participants’ accuracy on the learning assessment at the end of the learning task. We additionally computed accuracy during the flanker task based on the proportion of trials in which the participant correctly identified the direction of the central arrow. We also computed average response time (RT), excluding trials with inaccurate responses or those in which the response time was > 2 SD above the individual mean.

**Supplementary Table 1**

Bivariate correlations across engagement ratings, selective attention scores, and overall learning scores

|  | **Overall Satisfaction** | **Overall Efficacy** | **Overall Engagement** | **Overall Effort** | **Overall Difficulty** | **RT Cost Score** | **Overall Learning Score** |
| --- | --- | --- | --- | --- | --- | --- | --- |
| **Age** | .23* | .13 | .17 | .043 | .025 | -.17 | .19 |
| **Overall Satisfaction** | - | .56*** | .77*** | .41*** | .10 | -.02 | .27** |
| **Overall Efficacy** | - | - | .55*** | .55*** | -.22* | -.26** | .44*** |
| **Overall Engagement** | - | - | - | .54*** | .20* | -.10 | .19* |
| **Overall Effort** | - | - | - | - | .09 | -.15 | .31** |
| **Overall Difficulty** | - | - | - | - | - | -.02 | -.13 |
| **RT Cost Score** | - | - | - | - | - | - | -.17^†^ |

†p < .10; *p < .05; **p < .01; ***p < .001

**Supplementary Table 2**

Lesson content and assessment questions

| **Information Category** | | | | **Lesson Topic** | | | | **Assessment Question** |
| --- | --- | --- | --- | --- | --- | --- | --- | --- |
|  | | | Wugs | | Bliffs | Chuttles | Merbas |  |
| **Planet** | | | |  |  |  |  |  |
| Planet Name | | | Punden | | Glenna | Quinto | Charat | What planet do the [Alien Name] live on? |
| Planet Solar System | | | Maximus | | Cavum Nigrum | Spatium | Cometas | The [Alien Name] planet is located in the ___ solar system. |
| Planet Location | | | 5^th^ | | 3^rd^ | 4^th^ | 2^nd^ | [Alien Name] live on the ___ planet from the sun in their solar system. |
| **Weather** | | | |  |  |  |  |  |
| Major Biome | | | Tundra | | Tropical Rainforest | Temperate Grassland | Desert | The [Alien Name] planet is primarily made up of ___. |
| Temperatures | | | Below freezing | | Consistently  ~ 100F | Long warm season; short cold season | Drastic daily temperature changes | The [Alien Name] planet often experiences ___. |
| Precipitation | | | Snowfall ~ 10 inches/year | | Annual rainfall  > 100 in | Only during the warm season | Very little precipitation | Which weather pattern occurs on the [Alien Name] planet? |
| **Biology** | | | |  |  |  |  |  |
| Major adaptation | | Three fat layers to insulate against cold | | | Four-chambered stomach for digestion | Oil secretion for waterproofing | Enhanced red blood cells for water storage | The purpose of the [Alien Name] adaptation is to ___. |
| Temperature | | Low – 88F | | | High – 104F | Matched to external temperatures | Lower at night | The [Alien Name] body temperature is ___. |
| Skin | | Thick | | | Green | Scale-covered | Low pigment | The [Alien Name] have ___ skin to ___. |
| Internal Structure | | Dense, strong bones | | | Three lungs | Powerful saliva | Infrared vision | The [Alien Name] bodies have ___. |
| **Occupation** | | | |  |  |  |  |  |
| Primary Occupation | | Mining | | | Weaving | Farming | Building | What is the primary occupation of the [Alien Name]? |
| Primary Product | | Trilomite | | | Merrywick | Lykofruit | Scrib sand | What is the main product used by the [Alien Name]? |
| Product Step 1 | | Harvesting | | | Scavenging | Sprouting | Filtering | What is the correct order of the steps involved [Alien Name] work? |
| Product Step 2 | | Washing | | | Trimming | Bush growth | Mixing | As part of their work [Alien Name] complete/observe [step] in order to/when ___. |
| Product Step 3 | | Purification | | | Stem soaking | Flowering | Molding | As part of their work [Alien Name] complete/observe [step] in order to/when ___. |
| Product Step 4 | | Rapid cooling | | | Braiding | Fruiting | Baking | As part of their work [Alien Name] complete/observe [step] in order to/when ___. |
| Product Step 5 | | Sealing | | | Drying | Shedding | Construction | As part of their work [Alien Name] complete/observe [step] in order to/when ___. |
| Product Uses | | Heating, Cooking, Transportation | | | Clothing, Furniture,  Tents | Sweet porridge, Purple pie,  Roasted seeds | Homes, Stadiums, Tombs | [Alien Name] specifically use their product for all of the following except ___. |
| **Game** | | | |  |  |  |  |  |
| Common Game | Twiddledum | | | | Basket run | Seedles | Boulder racing | What is the name of the [Alien Name] favorite game? |
| Gameplay | Toss bean bag through hoop | | | | Carry ball in a basket | Drop seeds down a peg board | Push boulders around a track | The [Alien Name] favorite game is played by ___.    Which item is needed for the [Alien Name] to play their favorite game? |
| **Pet** | | | |  |  |  |  |  |
| Common Pet | Migworm | | | | Yelman | Beetlebird | Urvu gecko | What is the name of the [Alien Name] most common pet? |
| Animal Type | Invertebrate | | | | Mammal | Bird | Reptile | The [Alien Name] pets are ___. |
| Where They Live | Inside owner home | | | | In treetops | On rooftops | On bricks of owner home | The [Alien Name] pets live ___. |
| What They Eat | Blue algae | | | | Fruits and insects | Worms | Sand mites | The [Alien Name] pets eat ___. |
| Role in Alien Family | Cuddle | | | | Monitor for intruders | Learn to do tricks | Locate water | The [Alien Name] pets ___ with their owners. |

**Supplementary Table 3**

Duration of Instructional Videos for each Lesson Topic

| **Video Lesson Durations** | | | | | | |
| --- | --- | --- | --- | --- | --- | --- |
|  | |  | **Lesson Topic** | | | |
|  | **Overall**  **(Mean, SD)** | | **Wugs** | **Bliffs** | **Chuttles** | **Merbas** |
| Uncued – Narration | **4 min, 21 s**  **(5.9 s)** | | 4 min, 18 s | 4 min, 27 s | 4 min, 24 s | 4 min, 14 s |
| Cued – Narration | **4 min, 21 s**  **(7.1 s)** | | 4 min, 20 s | 4 min, 25 s | 4 min, 27 s | 4 min, 11 s |
| Uncued – Instructor Video | **4 min, 24 s**  **(6.7 s)** | | 4 min, 19 s | 4 min, 31 s | 4 min, 29 s | 4 min, 18 s |
| Cued – Instructor Video | **4 min, 25 s**  **(5.6 s)** | | 4 min, 21 s | 4 min, 27 s | 4 min, 32 s | 4 min, 20 s |
| **Overall Mean:** | **4 min, 22 s**  **(6.0 s)** | |  | | | |

**Note.** Timing differences across lesson topics were due to small differences in the number of spoken words per lesson, while timing differences within lesson topics (i.e., when lesson content was the same) were due to minor fluctuations in the rate at which the instructor spoke and the duration of pauses between slides of the presentation.

**Supplementary Table 4**

Engagement, Effort, and Difficulty Rating Scales

| **Rating Statement** | **Measure** |
| --- | --- |
| I enjoyed learning this way. | Satisfaction |
| I would like to learn this way in the future. | Satisfaction |
| I feel like I have a good understanding of the material. | Learning Efficacy |
| I felt like the instructor was working with me to help me understand the material. | Engagement |
| I found the instructor’s teaching style engaging. | Engagement |
| I felt motivated to try to understand the material. | Engagement |
| I felt that the subject matter was difficult. | Difficulty |
| Please rate the amount of effort that you put into understanding the material. | Effort |

**Supplementary Figure 1**

Response times during the flanker task


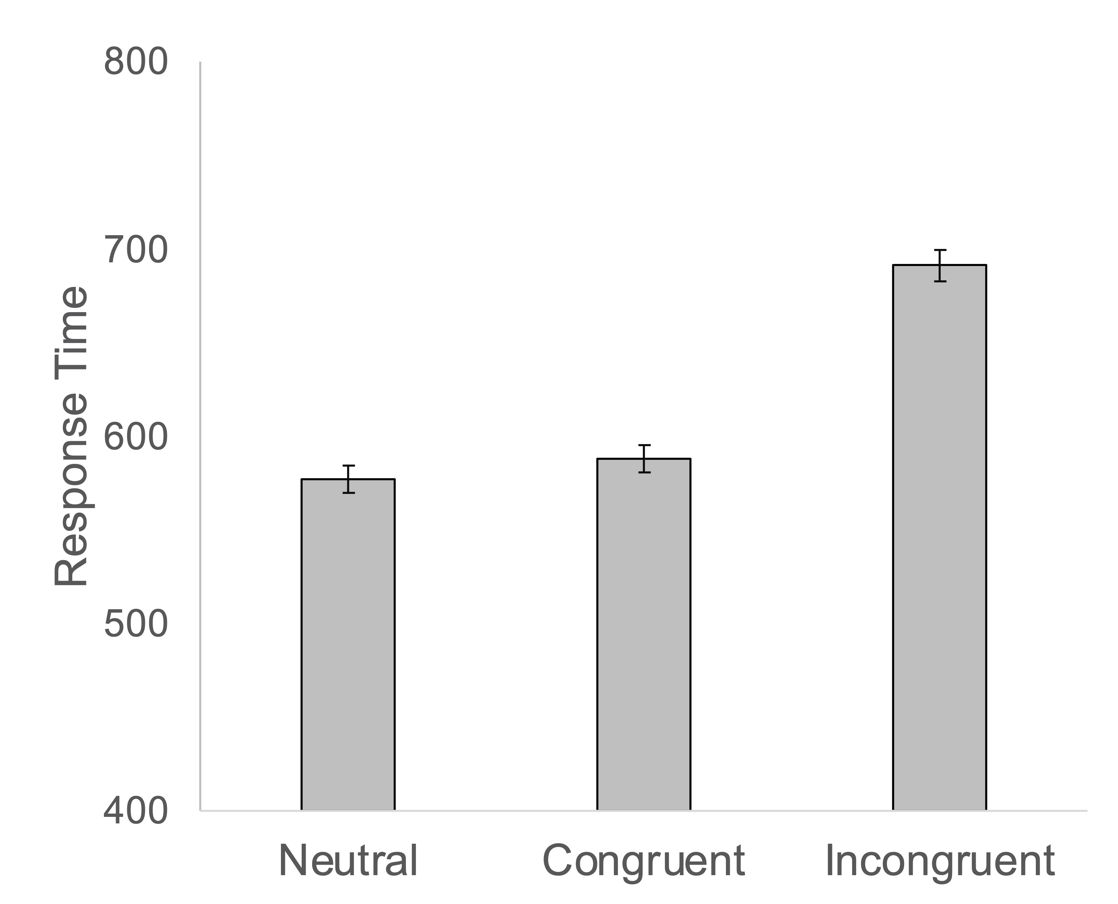

Supplement: Supplementary file 1 — Supplementary Information. [file 41598_2023_32069_MOESM1_ESM.docx]
